# MiR-181a Targets *RSPO2* and Regulates Bone Morphogenetic Protein – WNT Signaling Crosstalk During Chondrogenic Differentiation of Mesenchymal Stromal Cells

**DOI:** 10.3389/fcell.2021.747057

**Published:** 2021-10-29

**Authors:** Svitlana Melnik, Nina Hofmann, Jessica Gabler, Nicole Hecht, Wiltrud Richter

**Affiliations:** Research Center for Experimental Orthopaedics, Heidelberg University Hospital, Heidelberg, Germany

**Keywords:** miR-181a, mesenchymal stromal cells (MSC), chondrogenesis, osteoarthritis (OA), *RSPO2*, WNT signaling, BMP signaling, microRNA

## Abstract

Mechanisms of WNT and bone morphogenetic protein (BMP) signaling crosstalk is in the focus of multiple biological studies, and it also has been discovered to play important roles in human mesenchymal stromal cells (MSC) that are of great interest for neocartilage engineering due to their high chondrogenic differentiation potential. However, MSC-derived chondrocytes undergo hypertrophic degeneration that impedes their clinical application for cartilage regeneration. In our previous study, we established that several microRNAs (miRs) are differentially expressed between articular chondrocytes (AC) – and MSC-derived neocartilage, with miR-181a being the most prominent candidate as key microRNA involved in the regulation of a balance between chondral and endochondral differentiation. The aim of this study was the identification of precise mRNA targets and signaling pathways regulated by miR-181a in MSC during chondrogenesis. MiR-181a was upregulated during chondrogenesis of MSC, along with an increase of the hypertrophic phenotype in resulting cartilaginous tissue. By *in silico* analysis combined with miR reporter assay, the WNT signaling activator and BMP signaling repressor *RSPO2* was suggested as a target of miR-181a. Further validation experiments confirmed that miR-181a targets *RSPO2* mRNA in MSC. It was found that in human MSC miR-181a activated BMP signaling manifested by the accumulation of SOX9 protein and increased phosphorylation of SMAD1/5/9. These effects, together with the concomitant reduction of canonical WNT signaling induced by miR-181a mimic, were in accordance with the effects expected by the loss of *RSPO2*, thus indicating the causative link between miR-181a and *RSPO2*. Moreover, we observed that a tight correlation between miR-181a and miR-218 expression levels in healthy human cartilage tissue was disrupted in osteoarthritis (OA) highlighting the importance of the WNT-BMP signaling crosstalk for preventing OA.

## Introduction

Mesenchymal stromal cells (MSC) found in bone marrow and other tissues are an important class of adult stem cells capable of differentiation into various mesenchymal cell lineages (Friedenstein et al., 1987; Winter et al., 2003). Because of these properties, MSC are of great interest for regenerative medicine, and in particular, for treatment of musculoskeletal disorders, including osteoarthritis (OA). Once subjected to *in vitro* chondrogenesis, MSC undergo distinct stages of differentiation: from prochondrocytes that start to produce collagen type II, through chondroblasts characterized by upregulation of aggrecan (*ACAN*) expression, to mature chondrocytes, with high deposition of glycosaminoglycan (GAG) and proteoglycans, – a major component of extracellular matrix (ECM) of hyaline cartilage. However, unlike articular chondrocytes that remain restricted to a stable mature chondrocyte phenotype, MSC-derived chondrocytes proceed to a terminal stage of chondrogenesis and undergo hypertrophy characterized by an accumulation of collagen type X, activation of alkaline phosphatase (ALP) and matrix-metalloproteinase secretion (in particular, collagenases MMP13 and MMP3) that destroy ECM. As cartilage degradation in OA is accompanied by pathological changes of chondrocytes with activation of the hypertrophic phenotype, elucidation of the mechanisms that trigger chondrocyte phenotype instability and their progression into hypertrophy remains at the focus of cartilage biology research.

While articular cartilage neogenesis is controlled by the interaction of multiple cell signaling pathways: WNT (canonical and non-canonical), transforming growth factor β (TGF-β), bone morphogenetic protein (BMP), parathyroid hormone-related peptide (PTHrP), and Indian Hedgehog (IHH) (Goldring et al., 2006; Fischer et al., 2010; Dexheimer et al., 2016; Diederichs et al., 2019), it has been suggested that the WNT-BMP network is a major regulator of hypertrophy and cartilage-bone specification during chondrogenesis (Dexheimer et al., 2016; Diederichs et al., 2019). At the beginning of chondrogenesis, canonical WNT signaling is essential for chondrocyte proliferation and differentiation (Chen et al., 2008). It drives BMP signaling upregulation (Marcellini et al., 2012; Diederichs et al., 2019) that is essential for chondrogenesis (Dexheimer et al., 2016), and in particular, for expression of the SRY-Box Transcription Factor 9 (*SOX9*), a master regulator of chondrogenic differentiation (Akiyama et al., 2002; Lefebvre et al., 2019). However, once BMP signaling is activated, it synergizes with canonical WNT signaling in driving ALP activity (Rawadi et al., 2003; Itasaki and Hoppler, 2010), which contributes to hypertrophic degeneration of chondrocytes (Ishikawa et al., 1987; Mak et al., 2008). Indeed, application of WNT inhibitors during chondrogenesis lowered expression of hypertrophic markers, and this was accompanied with a decrease of BMP signaling, thus confirming that canonical WNT and BMP pathways cooperate in promoting the pro-hypertrophic differentiation (Diederichs et al., 2019). Inhibition of canonical WNT signaling, however, could not entirely block endochondral development *in vitro* and cartilage tissue ossification *in vivo* (Diederichs et al., 2019), and inhibition of BMP signaling completely abrogated chondrocyte differentiation (Dexheimer et al., 2016).

Altogether, this leads to the conclusion that an insufficiently understood WNT-BMP network regulates hypertrophy and chondral versus endochondral differentiation during chondrogenesis. Thus, more investigations on important players regulating their crosstalk are necessary to address underlying mechanisms.

It is still unclear which precise factors regulate the hypertrophic differentiation of chondrocytes. However, it has been established that microRNAs play an essential role in induction and progression of different stages of chondrogenic differentiation (Gabler et al., 2015).

MicroRNAs (miRs) are a class of small (∼ 22 nt) non-coding RNAs expressed in a cell- and tissue-specific manner and regulating a broad range of biological processes on post-transcriptional level by mediating mRNA degradation via direct binding to target mRNAs. In our previous studies, we addressed the question which miRs are involved in regulation of specific phases of chondrogenic maturation, especially hypertrophy (Gabler et al., 2015; Melnik et al., 2020). By miR expression array profiling done with human bone marrow MSC at different stages of chondrogenesis versus re-differentiated articular chondrocytes, we identified a distinct set of stage-specific miRs for the four populations of chondrocytes: prechondrocytes, chondroblasts, mature, and hypertrophic chondrocytes. We established the miR-181 family as the most prominent miR candidate that displayed differential expression between successive maturation stages of chondrocytes. The highly conserved miR-181 family consisting of miRs-181a/b/c/d allowed discrimination between four different differentiation stages of chondrocytes, with low expression levels in prechondrocytes, which was significantly enhanced in chondroblasts and chondrocytes, and further upregulated in hypertrophic chondrocytes. Additionally, a correlation between miR-181a expression and *COL10A1* levels found *in vitro* (Gabler et al., 2015) and *in vivo* (McAlinden et al., 2013) during chondrocyte differentiation implicated a putative role of miR-181a in regulation of hypertrophy. However, direct mRNA targets for miR-181a and mechanisms underlying its function during chondrogenesis remained unclear.

First discovered in 2006 (Guimaraes-Sternberg et al., 2006), the miR-181 family, primarily miR-181a, has become implicated in regulation of differentiation states of a wide range of biological processes and pathologies: cancer progression and cancer cell apoptosis (Feng et al., 2018; Braicu et al., 2019), insulin metabolism and diabetes mellitus (Kang et al., 2020; Cheng et al., 2021), T-cell differentiation and spontaneous autoimmunity disorders (Li et al., 2007; Schaffert et al., 2015), myocardial infarction and heart muscle function (Chen et al., 2018; Qi et al., 2020), and others. Functions of miR-181a have been extensively studied in T-cells, where it has been shown to dynamically regulate all stages of differentiation and maturation during the T-cell life cycle, from their development in the thymus to differentiation and eventually aging in the periphery. Functionally, e.g., in T-cells, miR-181a has been shown to target a number of phosphatases, in particular, dual specificity protein phosphatases 5 and 6 (*DUSP5* and *DUSP6*) that cause dephosphorylation of extracellular signal–regulated kinase (ERK) (Li et al., 2007). Thus, miRNA-181a is an important factor in cellular development and differentiation, evidenced by its aberrant expression in cancers and autoimmunity disorders (Calin and Croce, 2006; Lashine et al., 2011).

However, in many studies assessing the role of miR-181a in regulation of different pathologies, e.g., various cancers, there is lack of clear consensus whether miR-181a functions as a tumor suppressor or, in contrary, it contributes to tumor progression (Seoudi et al., 2012). Similarly to this, in the musculoskeletal field there have been some discrepancies in evaluating the functions of miR-181a. Only a few functional studies on the role of miR-181a in chondrogenesis are available to date. On one hand, they have reported that miR-181a mediates cartilage degeneration (Nakamura et al., 2016), and attenuation of miR-181a expression could be used as a therapeutic approach in treating patients with OA in facet and knee joints (Nakamura et al., 2019). Additionally, a recent study with adipose-derived mesenchymal stem cells subjected either to adipogenic or osteogenic differentiation reported about significant enhancement of osteogenesis upon gain of miR-181a delivered with poly(lactic-co-glycolic acid) (PLGA) nanofibers (Qi et al., 2021). On the other hand, a different study has demonstrated that miR-181a promotes early stages of chondrogenesis as its depletion significantly inhibited chondrogenic differentiation in pig peripheral blood MSC (Zhao et al., 2018). However, the mechanistic details on modulation of chondrogenic differentiation with miR-181a, as well as its direct targets remained unclear.

In the present study, we addressed the question which role in MSC chondrogenesis does miR-181a play: whether it promotes or suppresses chondrogenic differentiation. For this, we identified a precise mRNA target for miR-181a, as well as interrogated its effects on the signaling pathways driving MSC chondrogenesis and hypertrophy. A better understanding of mechanisms involved in chondrogenic differentiation will contribute to the development of new approaches that allow manipulation of MSC differentiation outcome for tissue engineering and to new intervention strategies against OA pathology progression in patients.

## Materials and Methods

### Cell Lines, Isolation of Mesenchymal Stromal Cells and Articular Chondrocytes, Cell Expansion

Human embryonic kidney epithelial HEK293T cells (ATCC CRL-3216^TM^) were grown in DMEM (Gibco) supplied with 10% fetal calf serum (FCS, Sigma), 100 I.U./mL penicillin, 100 μg/mL streptomycin (Biochrome).

MSC were isolated from fresh bone marrow aspirates of patients undergoing elective bone surgery [*N* = 23 donors, median age 59 (range 38–75), 14 females/11 males]. AC were isolated from knee cartilage of *tibia plateau* that was removed due to OA (*N* = 15 donors), or from healthy donors (*N* = 20), either undergoing arthroscopic surgery of a knee due to trauma, or deceased due to unrelated causes. Healthy articular cartilage was carefully removed from regions with no macroscopically evident degeneration and washed with phosphate buffered saline (PBS), to avoid contamination by other cells. To assess the severity of OA in each patient, the International Cartilage Repair Society (ICRS) scoring system (ranging from 0, 1a-b, 2, 3a-b-c-d, and 4) was applied (Kleemann et al., 2005). All the OA patients had the same highest score 4 (Supplementary Table 1).

The study was approved by the local Ethics Committee (Medical Faculty of the University of Heidelberg), and informed consent was obtained from all the participating patients, according to the 1964 Declaration of Helsinki, updated in 2000. All cells used for experiments in the study were from HIV-, HBV-, and HCV-negative donors.

MSC were isolated, as described before (Dexheimer et al., 2016). In brief, the mononuclear cell fraction was separated from bone-marrow aspirates by Ficoll-Paque^TM^ density-gradient and seeded at density of 1.25 × 10^5^ cells/cm^2^ into 0.1% gelatin-coated culture flasks in expansion medium [DMEM high glucose, 12.5% FCS, 100 I.U./mL penicillin, 100 μg/mL streptomycin, 2 mM L-glutamine, 1% non-essential amino acids, 0.1% β-mercaptoethanol (all from Life Technologies), 4 ng/mL FGF-2 (Active Bioscience)]. After 24 h, non-adherent cells were removed by washing with PBS. Medium was replaced three times a week, and cells were expanded until passage 3. For passaging, once cells reached 80% of confluence, they were detached with trypsin/EDTA and plated at density 5000 cells/cm^2^.

For AC isolation, cartilage pieces were minced, and cells were released by overnight by digestion with collagenase B (Roche) and hyaluronidase (Sigma-Aldrich), as described before (Bothe et al., 2018). AC were expanded in DMEM medium containing 10% FCS, 100 I.U./mL penicillin, 100 μg/mL streptomycin, at 37°C, 6% CO_2_. Medium was changed twice per week, and cells were expanded until passage 2.

### Chondrogenic Differentiation and Re-differentiation

For chondrogenic differentiation (MSC) or re-differentiation (AC), at 3rd (MSC) or 2nd (AC) passages, 5 × 10^5^ MSC or AC were resuspended in chondrogenic medium [DMEM high glucose, 0.1 μM dexamethasone, 0.17 mM ascorbic-acid 2-phosphate, 5 mg/ml transferrin, 5 ng/ml sodium selenite, 1 mM sodium pyruvate, 0.35 mM proline, 1.25 mg/ml bovine serum albumin (BSA), 100 I.U./mL penicillin, 100 μg/mL streptomycin, 5 mg/ml insulin (Sanofi-Aventis), 10 ng/ml TGF-β1 (from PeproTech or BPS Bioscience)]. Cells were centrifuged at 500 × *g* for 5 min, or allowed to self-aggregate without centrifugation, to generate high-density pellets. Pellets were cultured for 3, 4, or 6 weeks at 37°C, 6% CO_2_ with medium change three times a week.

### *In silico* Target Prediction Analysis and Kyoto Encyclopedia of Genes and Genomes Pathway Analysis

MicroRNA target prediction web-based software tools TargetScan^1^, miRWalk^2^, and miRanda^3^ were applied to identify putative mRNA targets. Putative mRNA targets with strong mirSVR (microRNA score vector regression) downregulation score (-0.4)^4^ suggested by all used prediction tools were included in further consideration and analysis. For systematic analysis of gene Ontology (GO) terms and Kyoto Encyclopedia of Genes and Genomes (KEGG) signaling pathways of putative mRNA targets suggested by the miR prediction tools, the Database for Annotation, Visualization, and Integrated Discovery (DAVID) gene functional classification tool was applied, with a stringency set at the highest level (Dennis et al., 2003).

### RNA Extraction, Quantitative Reverse-Transcriptase PCR

Total RNA was isolated from cells and pellets using a standard guanidinium thiocyanate/phenol extraction protocol with peqGOLD TriFast^TM^ reagent (Peqlab). Total RNA from cartilage tissue was isolated, as described before (Le Bleu et al., 2017; Hecht et al., 2019). Polyadenylated mRNA was isolated using oligo(dT)-coupled magnetic beads (Dynabeads, Dynal, Invitrogen), according to the manufacturer’s instruction. For the first strand cDNA synthesis, 20 ng of mRNA were used with reverse transcriptase (OmniScript^®^, Qiagen) and oligo(dT) primers. Quantitative reversed transcriptase PCR (qRT-PCR) was performed using SYBR green I mix (Thermo Fisher Scientific) and gene-specific primers with Stratagene Mx3000P (Agilent Technologies).

For quantification of microRNA expression, 10 ng of total RNA were used for cDNA synthesis with the TaqMan^®^MicroRNA Reverse Transcription Kit (Thermo Fisher Scientific). Relative expression levels were determined by qRT-PCR using the miR detection method from Applied Biosystems (MicroRNA Reverse Transcription Kit) combined with the TaqMan MicroRNA Assays, using primers for U6, hsa-miR-218-5p and has-miR-181a from Thermo Fisher Scientific (Assay IDs: 0001973, 000521, and 000480, respectively). Expression levels of mRNA and miR were calculated using 2-ΔΔCT method (Rao et al., 2013) and normalized to housekeeping genes Actin Beta (*ACTB*), Hypoxanthine-guanine phosphoribosyltransferase (*HPRT*), Ribosomal Protein L13 (*RPL13*), and Cleavage and Polyadenylation Specific Factor 6 (*CPSF6*) (for mRNA), or U6 (Small Nuclear Ribonucleoprotein U6, *SNRNU6)* (for miR). All primers used for the assay were listed before (Melnik et al., 2020). For monitoring expression of human *RSPO2* (NM_178565.4) the following primers were used: forward 5′TGTCCAACCATTGCTGAATC3′ and reverse 5′TCCTCTTCTCCTTCGCCTTT3′, and human *KLF15* (NM_014079) was measured using forward 5′AGCCGCAG AACTCATCAAAA3′ and reverse TTGATGTGCTTGGAGA GGTG.

### 3′UTR Luciferase Reporter Assay

The 3′UTR of *RSPO2* containing a seed region for miR-181a was amplified from genomic DNA by PCR. The 3′UTR fragment was cloned into *Spe*I and *Hin*dIII restriction sites of pMIR-REPORT Luciferase vector (Thermo Fisher Scientific). The resulting plasmid containing wild type 3′-UTR of *RSPO2* subsequently was used as a template to generate a plasmid with mutated 3′-UTR of *RSPO2* lacking a seed region using Gibson Assembly. The 3′UTR of *KLF15* containing a seed region for miR-181a, as well as its corresponding mutated sequence, were chemically synthesized (GeneArt, Germany). Primers used for the assay are listed in Supplementary Table 3.

For miR gain-of-function experiments, microRNA mimics (non-targeting control NC and miR-181a) were purchased from Ambion (mirVana: 4464058, MC11141, respectively).

HEK293T cells, seeded a day before transfection either at a density 5 × 10^4^ cells/well, were cultured on 24 well plates (in case of *KLF15*), or at density 2 × 10^4^ cells/well on 96 well plates (in case of *RSPO2*). Co-transfection of 50 nM of a selected miR mimic combined either with 250 ng of a corresponding tested reporter construct, together with 250 ng of a normalization control β-Gal vector (Thermo Fisher Scientific) (in case of *KLF15*), or with 45 ng of a corresponding tested reporter construct, together with 5 ng of a *Renilla* –expressing plasmid pRL-TK (Promega) served as a normalization control, was carried out using Lipofectamine 2000 reagent (Invitrogen, Germany), or X-tremeGENE 9 (Roche). Luciferase activity was measured 48 h after transfection with Victor3 Multilabel Counter 1420–042, or with FLUOStar Omega (BMG LABTECH) using the Luciferase Reporter Assay, or Dual Luciferase Assay kits (Promega). Luciferase intensity signals were normalized to the β-Galactosidase activity or *Renilla* luciferase signal in the same sample. Three independent experiments with six biological replicates were performed for this assay.

### Ectopic Overexpression of miR-181a in Mesenchymal Stromal Cells

For miR overexpression in MSC cells, a reverse method of transfection using DharmaFECT 1 (Dharmacon) was carried out, according to the manufacturers’ instruction. In brief, 10^6^ MSC at passage 3 resuspended in 100 μl of DMEM medium containing no additives were transfected with 50 nM of miR mimic, according to the manufacturer’s protocol. The transfected cells were transferred in chondrogenic medium and subjected to chondrogenic differentiation for 28 days, as described above.

### Western Blotting

Cells or cartilaginous tissue pellets were lysed in lysis buffer containing 50 mM Tris–HCl (pH 7.4), 150 mM NaCl, 1% Triton X-100 and 1:100 Halt Protease and Phosphatase Inhibitor Cocktail (Thermo Fisher Scientific) for 5 min on ice, with a preliminary grinding step (in case of pellets) using Powerful ball mill Retsch MM400 (2 rounds for 2 min, 30 Hz). The lysates were cleared by centrifugation, and proteins were resolved by SDS-PAGE, blotted onto a nitrocellulose membrane and analyzed by WB. Following antibodies were used: phospho-SMAD1/5/9 (Cell Signaling, 13820), total SMAD1/5 (Abcam ab33902 and Abcam ab40771), SOX9 (Millipore AB5535), KLF15 (Santa Cruz sc-393627), phospho-ERK1/2 (Santa Cruz sc-7383), total ERK1/2 (Cell Signaling #9102), β-catenin (BD Transduction Laboratories, 610153), active β-catenin (Merck Millipore 05-665), and α-tubulin (Thermo Fisher Scientific, MS-581-P0).

### Histology and Immunohistochemistry

Pellets were fixed for 24 h in 4% formaldehyde, dehydrated in a graded isopropanol series and paraffin-embedded. Thin sections (5 μm) were stained with Safranin O (0.2% in 1% acetic acid) using Certistain^®^ fast green (0.04% in 0.2% acetic acid) for counter-staining, according to a standard procedure. Immunohistochemical detection of collagens, type II and type X, was performed as described (Winter et al., 2003). Briefly, sections treated with 4 mg/mL hyaluronidase in PBS, pH 5.5 and 1 mg/mL pronase, then blocked with 5% BSA and stained with collagen type II antibody (clone II-4C11, ICN Biomedicals), or collagen type X antibody (clone X53, Quartett, Germany), followed by incubation with biotinylated goat anti-mouse antibody (1:500, Dianova), and subsequent detection with streptavidin alkaline-phosphatase fast red (Thermo Fisher Scientific).

### Alkaline Phosphatase Activity Assay

Alkaline phosphatase activity was measured by colorimetric assay using multi-mode plate reader FLUOStar Omega (BMG LABTECH). For this, cell culture supernatants (5 per group or treatment), conditioned for 2 days, were collected and mixed with an equal volume (100 μl) of ALP substrate solution (10 mg/ml p-nitrophenylphosphate in 0.1 M glycine, 1 mM MgCl_2_, and 1 mM ZnCl_2_, pH 9.6). After incubation for 180 min, absorbance recorded at 405 nm was corrected for a signal at 490 nm, and enzyme activity was quantified using a standard curve built using serial dilutions of p-nitrophenol (Sigma-Aldrich). Six independent experiments with two technical replicates were performed for this assay.

### Quantification of Proteoglycan Content (Dimethylmethylene Blue Assay)

Proteoglycan content in cartilaginous tissue was measured by dimethylmethylene Blue (DMMB) assay, as described before (Melnik et al., 2019), and the values were normalized to DNA amount in lysed cells measured with Quant-iT PicoGreen dsDNA kit (Invitrogen, Eugene, United States). For this, 20 μl of the digested pellet sample were mixed with 80 μl TE buffer (200 mM Tris HCl, 20 mM EDTA) and PicoGreen solution, and fluorescence in samples was measured at 485/535 nm.

### Statistical Analysis

Statistical analysis was performed using GraphPad (Prism) software, with application of Mann–Whitney *U*-Test, or two-way ANOVA Bonferroni’s Multiple Comparison Test, or paired two-tailed Student’s *t*-test, or Pearson correlation test; *p*-values ≤ 0.05 were referred to as being significant.

## Results

### Differential Expression of miR-181a Between Mesenchymal Stromal Cells and Articular Chondrocytes During Differentiation Correlated With the Hypertrophic Phenotype

In our previous studies using microRNA microarray analyses with application of 1349 miR probes, we identified top 15 differentially expressed miRs that reached over 60-fold changes between MSC that underwent hypertrophic differentiation versus AC that retained the stable mature chondrocyte phenotype during an *in vitro* chondrogenesis time-course in 3D pellet cultures (Gabler et al., 2015; Melnik et al., 2020). The most striking differential expression profiles were observed for the miR-181 family that also correlated with the progression of the successive maturation stages of chondrogenesis. Among the four miR-181 family paralogs, miR-181a was the most significantly overrepresented miR in the microarray data (Supplementary Figure 1). Thus, miR-181a was selected for further analyses.

**FIGURE 1:**
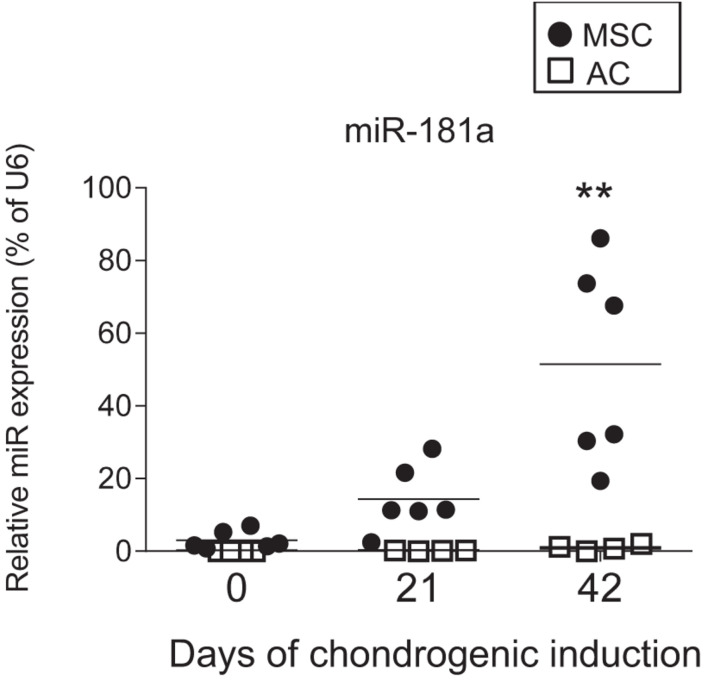
Differential expression of miR-181a between MSC (*N* = 6 donors, black circles) and AC (*N* = 4 donors, white squares) was monitored at indicated time points during chondrogenic differentiation by qRT-PCR; vertical lines correspond to median values; **p ≤ 0.001 (Two-way ANOVA).

Next, we validated the microarray data using qRT-PCR to monitor the expression levels of miR-181a in chondrocytes derived from MSC versus AC over the time-course of chondrogenesis. We found that at the start of differentiation, miR-181a had very low expression levels both in AC and MSC. However, it was significantly upregulated at later time points (days 21 and 42) in MSC, but, not in AC, where it remained at low levels in each of the tested donors (Figure 1). Of note, these differences correlated with the outcome of chondrogenic differentiation observed in MSC- versus AC- derived neocartilage tissue pellets. In particular, both tissues showed pronounced staining with Safranin O and comparable levels of collagen type II immunostaining. However, a positive immunostaining for the hypertrophic marker collagen type X was present only in MSC-derived pellets, whereas it was completely negative in AC (Supplementary Figure 2A), indicating that MSC, but not AC, underwent hypertrophy. This conclusion was additionally supported by the expression data for *COL10A1/COL2A1*, which highlighted upregulation of the hypertrophy-associated marker *COL10A1* exclusively in MSC (Supplementary Figure 2B). Collectively, a positive correlation between miR-181a and *COL10A1* expression indicates that miR-181a might be involved in regulation of chondrogenic differentiation, and in onset of the hypertrophic phenotype, in particular.

### *RSPO2* Is a Target of miR-181a

To identify putative targets of miR-181a, we applied three microRNA target prediction tools: TargetScan, miRWalk, and miRanda. This *in silico* analysis resulted in a list of 6615 high-confidence hits for miR-181a. Gene Ontology and KEGG pathway analyses revealed that among the found putative targets there was a significant enrichment for genes related to “Signal transduction” (including known chondrogenesis-regulating signaling pathways, e.g., TGF-β, WNT, and PTHRP) (Supplementary Table 2). Altogether, this suggested a rather high complexity of putative miR-181a regulation mechanisms.

Next, we selected those putative gene candidates that had a significant mirSVR score. This score was computed by the microRNA.org tool that uses vector regression (SVR) to evaluate on a wide range of miR features allowing prediction and ranking of multiple putative microRNA target sites’ efficiencies. MirSVR score serves as a measure of the likelihood that a given miR interacts with a certain sequence of mRNA depending on a secondary structure accessibility of the site and its conservation that affects miR-mRNA pairing. A score below (- 0.4) is generally regarded as significant. Two genes, *KLF15* and *RSPO2*, were selected based on this approach (Table 1), as well as on their relevance to chondrogenic differentiation established on the available to date literature data.

**TABLE 1:** *In silico* analysis for annealing efficiencies between putative mRNA targets and miR-181a.

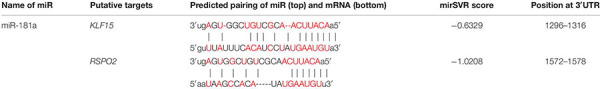

*Paired nucleotides are depicted in red.*

In particular, Krüppel-like transcription factor 15 (*KLF15*), has been suggested as a novel factor involved in regulation of chondrogenesis and OA development (Li et al., 2018). KLF15 was demonstrated to play an important role in chondrogenic differentiation in a time-dependent manner by activating the expression of *SOX9* via direct binding and promoter initiation (Song et al., 2017). It also has been shown to directly suppress expression of the Matrix metalloproteinase-3 (*MMP3*) gene. High levels of KLF15 were found in primary chondrocytes from healthy donors, with significant decline in chondrocytes from OA patients (Li et al., 2018), therefore, implicating a regulatory role of KLF15 in hypertrophic phenotype development.

The other selected gene, *RSPO2*, a member of the R-spondin gene family, encodes a secreted protein that is known to be a strong canonical WNT agonist (Kazanskaya et al., 2004; Glinka et al., 2011). At the same time, RSPO2 has functions beyond its role in the amplification of canonical WNT signaling as it acts as BMP4 signaling inhibitor (shown in *Xenopus* and in the human hepatocarcinoma cell line HepG2) (Lee et al., 2020). Interestingly, RSPO2 has been implicated in hypertrophy and OA development by a number of studies. In our previous study (Diederichs et al., 2019), aiming to identify factors related to hypertrophic MSC differentiation, *RSPO2* was among the most differentially expressed genes between hypertrophic and non-hypertrophic chondrocytes. Additionally, elevated expression of *RSPO2* (Takegami et al., 2016; Okura et al., 2019) was found to correlate with the levels of hypertrophy of chondrocytes in OA cartilage of human patients, thus indicating its putative role in the regulation of the pathologic phenotype during OA development. Inhibition of RSPO2 either with a neutralizing antibody or by treatment with the mianserin drug (Okura et al., 2019) has been shown to reduce cartilage degradation in a murine OA model. In turn, ectopic application of RSPO2 protein showed a drastic increase in hypertrophic chondrocyte differentiation and cartilage degradation in a murine OA model (Zhang et al., 2018). RSPO2, therefore, has been proposed as a putative therapeutic target for OA modulation.

Collectively, these studies indicate that both *KLF15* and *RSPO2* are involved in regulation of MSC chondrogenesis and might be linked to miR-181a functionally as its putative gene targets.

**FIGURE 2:**
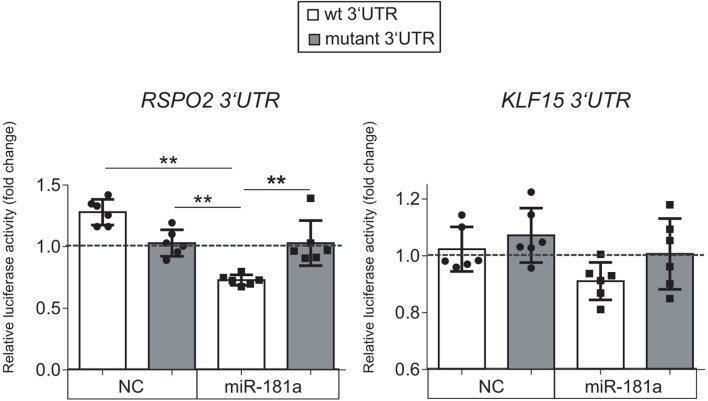
Luciferase reporter assay done in HEK293T cells with pMIR-REPORT vector either containing the wild-type (wt, white) or a mutated 3′-UTR (gray) fragment of the indicated mRNA as a putative binding site for miR-181a. The control reporter was containing the binding site for a non-targeting control miR (NC). The gray dotted line indicates levels of binding for an empty pMIR-REPORT vector; *n* = 6; error bars correspond to mean values ± SD; **p ≤ 0.01 (Two-way ANOVA).

Of note, low levels of miR-181a expression were gradually rising during MSC chondrogenesis (Figure 1), while expression of *KLF15* (Supplementary Figure 3) and *RSPO2* (see below) both remained at low levels. However, in AC, a reversed pattern was observed, with miR-181a expression remaining at low levels throughout the differentiation course (Figure 1 and Supplementary Figure 3) that was accompanied by upregulation of *KLF15* expression (Supplementary Figure 3) and constantly high levels of *RSPO2* (Supplementary Figure 4). This therefore implies that if miR-181a targets both molecules, increasing levels of miR-181a in MSC might have prevented the *RSPO2/KLF15* upregulation while low miR-181a expression in AC allowed it. To verify whether miR-181a can target *KLF15* or *RSPO2* mRNAs for degradation, we tested interactions between miR-181a and 3′UTRs of the corresponding genes in a reporter assay. It was found that miR-181a significantly reduced the activity of a reporter consisting of a wild-type *RSPO2* 3′UTR, with no effect in case of a mutant 3′UTR sequence, or when a non-targeting miR was used as a negative control of the miR mimic (Figure 2). In case of *KLF15*, we found that although there was some reduction of the reporter activity containing a wild-type 3′-UTR of *KLF15*, this effect was not significant. Thus, we hypothesized that miR-181a can target *RSPO2* mRNA for degradation. Of note, low levels of expression of miR-181a in AC during re-differentiation (Figure 1) were accompanied with steadily high levels of *RSPO2* (Supplementary Figure 4), in line with the hypothesis.

Collectively, these results indicated that *RSPO2*, and not *KLF15*, can be a primary target for miR-181a in the context of human MSC chondrogenesis.

### Gain of miR-181a Promoted Mesenchymal Stromal Cells Chondrogenesis by Activation of Bone Morphogenetic Protein Signaling

**FIGURE 3:**
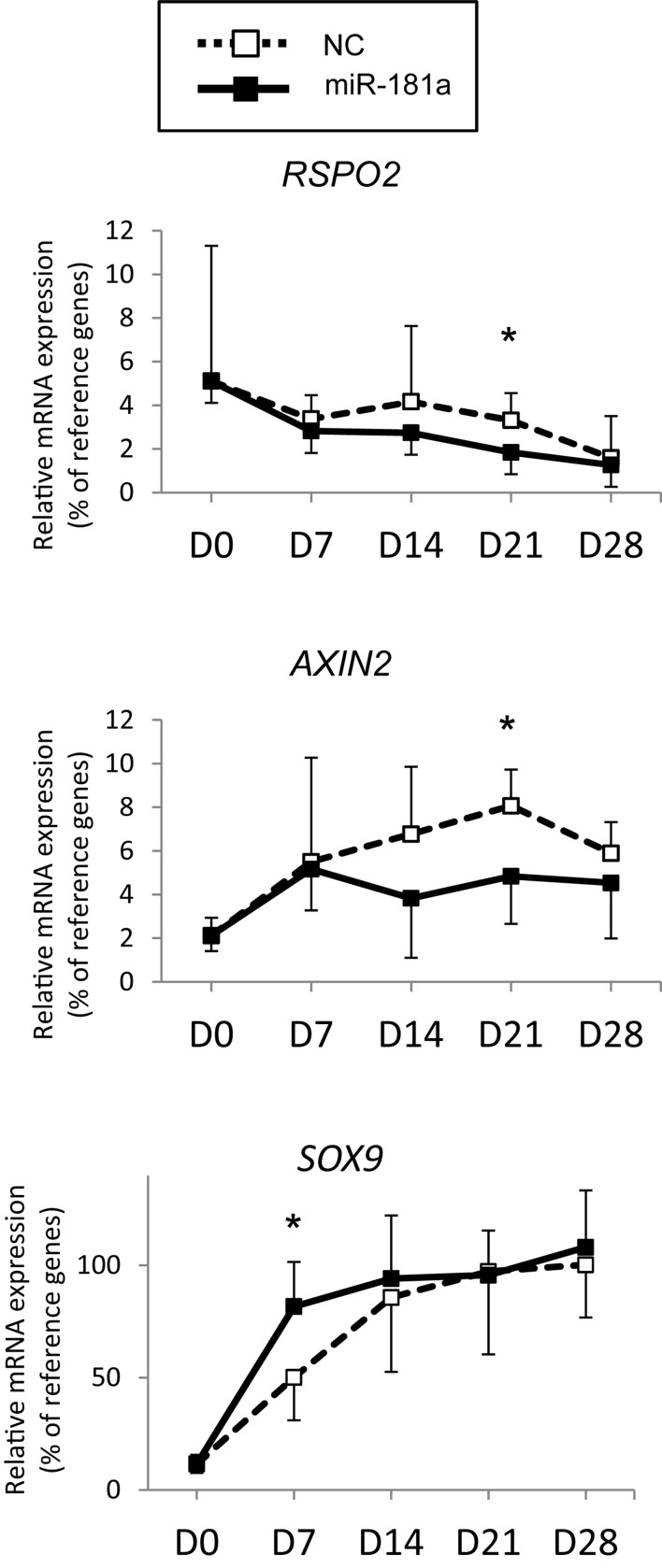
MSC (*N* = 5–6 donors) were transfected with miR-181a (black line, black squares), or with a control non-targeting miR (NC, black dotted line, white squares) mimic at day 0, then subjected to chondrogenic differentiation for 28 days, and mRNA expression for indicated genes was monitored at indicated time points by qRT-PCR. Levels of mRNA expression were normalized to a geomean of *HPRT, CPSF6* and *RPL13*. Error bars correspond to mean values ± SD; *p ≤ 0.05, NC vs miR-181a at indicated time points (Mann–Whitney *U*).

Next, assuming that miR-181a targets *RSPO2* in MSC, gain of miR-181a was expected to result in reduction of *RSPO2* expression. This should then translate into possible effects on WNT and BMP signaling as a consequence. To address the functional role of miR-181a in the context of MSC chondrogenesis and verify our hypothesis, we transfected the cells with miR-181a mimic to follow specific markers for BMP and WNT signaling activation or inhibition. First, we confirmed that significantly elevated levels of miR-181a mimic persisted at least over the 21 days time-course of chondrogenic differentiation (Supplementary Figure 5). This also caused a significant reduction of *RSPO2* expression by day 21 in MSC transfected with miR-181a in comparison to NC mimic control (Figure 3). Of note, by the end of chondrogenesis at day 28, levels of *RSPO2* expression leveled up between the treatments. Therefore, the effects caused by the transient gain of miR-181a achieved with our approach might last no longer than 21 days.

**FIGURE 4:**
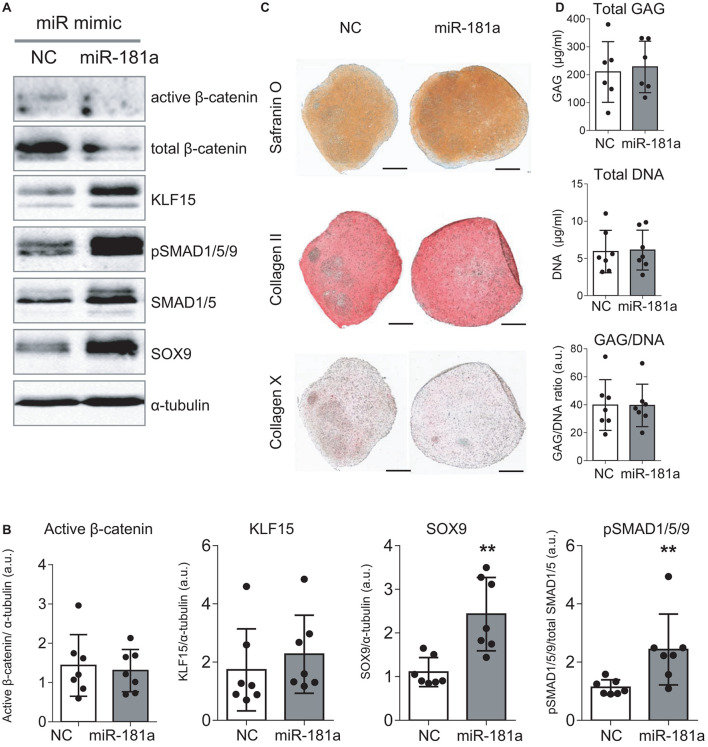
Gain of miR-181a at the start of chondrogenic differentiation boosted MSC chondrogenesis according to enhanced BMP signaling and SOX9 induction. MSC (*N* = 5–7) were transfected with miR-181a (gray), or with a control non-targeting miR (NC, white) mimic at day 0, and subjected to chondrogenic differentiation for 28 days. **(A)** Accumulation of the indicated proteins was assessed by Western blot analysis at day 28; α-tubulin was used as a loading control; **(B)** Semi-quantitative analysis of WB images; Error bars represent mean values ± SD; *N* = 7; **p ≤ 0.01 (Mann-Whitney *U*); **(C)** Safranin O staining and immunohistochemistry analysis of COLII and COLX in 3D pellets of the same donor shown in **(A)** at day 28 of chondrogenesis. Scale bar, 500 μm. **(D)** GAG/DNA content of 3D pellet cultures was assessed by DMMB assay at day 28 of chondrogenesis; error bars correspond to mean values ± SD; *N* = 7 donors.

As no antibodies for RSPO2 suitable for Western Blot application are available at present, we could not verify the effects of miR-181a on RSPO2 protein accumulation. Nevertheless, the observed effects on WNT and BMP signaling were in line with a probable loss of *RSPO2*. In particular, we found that expression levels of *AXIN2*, *a bona fide* target gene for canonical WNT signaling, were significantly reduced by day 21 (Figure 3), although no consistent effect was obvious for active β-catenin levels detected at day 28 (Figures 4A,B and Supplementary Figure 6). Of note, no consistent effects were detected for the total β-catenin as well. The total β-catenin mostly comprises of the membrane-bound fraction of β-catenin which has been shown to decline during chondrogenesis due to changes to the adhesion properties of cells and ECM deposition (Zhang and Stott, 2004). Remarkably, this trend for reduction of canonical WNT signaling was accompanied with activation of BMP signaling, manifested by a significant increase in phosphorylation of SMAD1/5/9 (Figures 4A,B and Supplementary Figure 6), as well as a significant increase in *SOX9* expression by day 7 (Figure 3), also observed at day 28 on the protein level (Figures 4A,B and Supplementary Figure 6). Of note, another known target gene of BMP signaling, *ID1*, was also upregulated by gain of miR-181a (Supplementary Figure 7).

Importantly, even though overall histological examination of the resulting cartilaginous pellets at day 28 of chondrogenesis pointed toward enhanced Safranin O staining in the miR-181a group (Figure 4C and Supplementary Figures 7,8) indicating higher proteoglycan deposition due to the treatment, no change in GAG/DNA content was obvious compared to the control treatment group (Figure 4D). Immunostaining for collagen type II and hypertrophy-related collagen type X was similar in both groups (Figure 4C) and this was also confirmed by expression data for *COL2A1* and *COL10A1* genes (Supplementary Figure 7). Nevertheless, a reduced ALP activity was detected in culture medium supernatants of the pellets of the miR-181a group (Supplementary Figure 9), in line with a trend for lower expression levels of the corresponding gene *ALPL* (Supplementary Figure 7). Additionally supported by a reduction of *MMP3* expression (Supplementary Figure 7), these data overall indicate that it might be possible to affect the balance between chondral versus endochondral differentiation by gain of miR-181a. It is normally expected that upregulation of BMP signaling would enhance ALP activity and increase hypertrophy as a consequence (Dexheimer et al., 2016). However, in case of miR-181a gain, we observed that the trend for concomitant WNT reduction might neutralize these effects. Thus, altogether, ALP activity and other hypertrophy-associated markers remained either unchanged or displayed a trend for reduction.

Collectively, these data support our hypothesis that *RSPO2* could be a target gene of miR-181a during MSC chondrogenesis. Moreover, they demonstrated an ability of miR-181a to alter the balance between two critical cell signaling pathways, WNT and BMP that regulate the outcome of MSC chondrogenesis.

### MiR-181a Inhibited ERK1/2 Phosphorylation in Mesenchymal Stromal Cells Chondrogenesis

The most comprehensive functional studies on the role of miR-181a were done in T-cells, where it was demonstrated that T-cell receptor sensitivity and signaling strength could be modulated at the posttranscriptional level by miR-181a (Li et al., 2007). In T-cells, gain of miR-181a resulted in a multiple tyrosine phosphatase inhibition, including *DUSP5* and *DUSP6*. This caused significant increase in kinase ERK1/2 phosphorylation and greater T-cell sensitivity, suggesting that miR-181a acts as an intrinsic antigen sensitivity rheostat during T-cell development.

**FIGURE 5:**
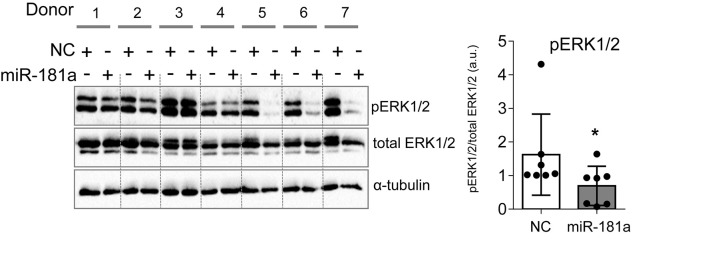
MiR-181a inhibited ERK1/2 phosphorylation in MSC chondrogenesis. MSC (*N* = 7) were transfected with miR-181a (gray), or with a control non-targeting miR (NC, white) mimic at day 0, and subjected to chondrogenic differentiation for 28 days. Accumulation of the indicated proteins was assessed by Western blot analysis at day 28 and a semi-quantitative evaluation of WB images for pERK1/2 was done in relation to total ERK1/2; α-tubulin was used as a loading control; error bars represent mean values ± SD; *p ≤ 0.05 (Mann–Whitney *U*).

We wondered whether gain of miR-181a would cause enhanced levels of ERK1/2 phosphorylation due to a similar mechanism. Surprisingly, we found that there was no increase in phospho-ERK1/2 upon miR-181a mimic transfection. Rather opposite, pERK1/2 levels were significantly reduced due to gain of miR-181a at day 28 of chondrogenesis (Figure 5). Therefore, it was unlikely that miR-181a utilized the same mechanism in MSC as in T-cells by targeting multiple phosphatases, and *DUSP5* and *DUSP6*, in particular.

### There Were No Differences in miR-181a Expression in Healthy Versus OA Cartilage, However, a Tight Correlation Between miR-181a and miR-218 in Healthy Cartilage Tissue Was Lost in OA

**FIGURE 6:**
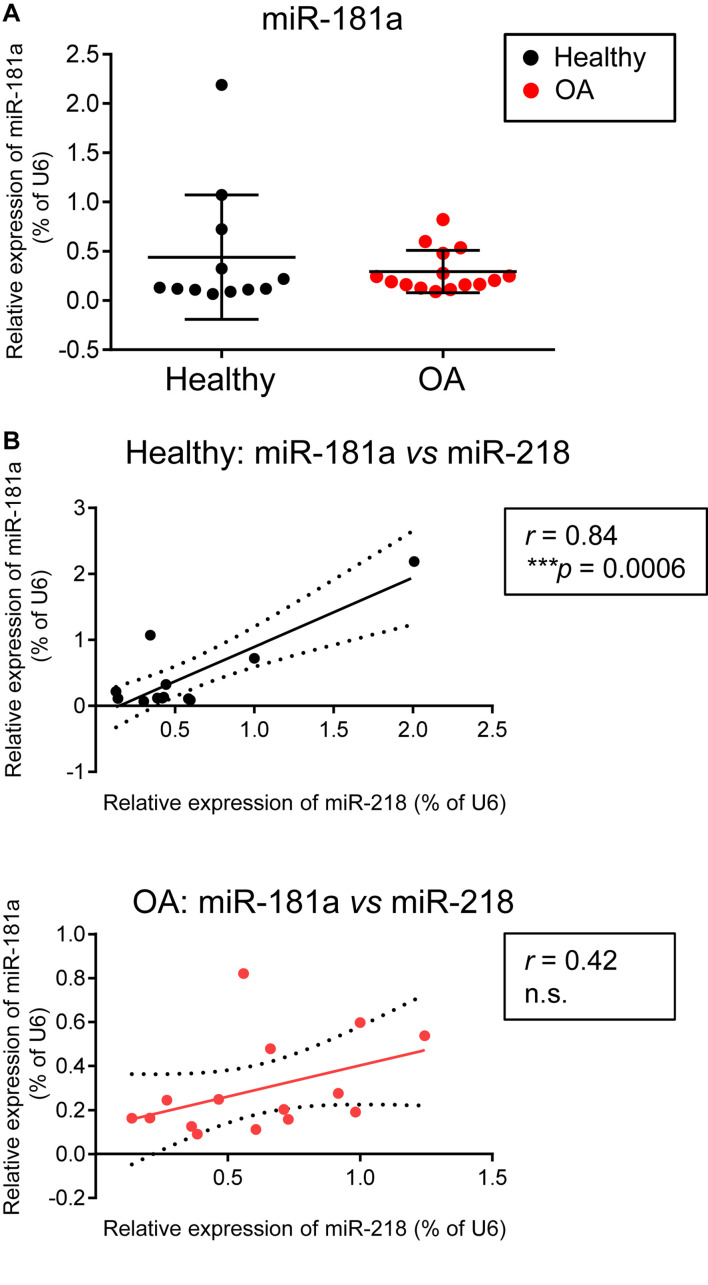
There were no differences in miR-181a expression levels between healthy and OA cartilage, however, a tight correlation between miR-181a and miR-218 in healthy cartilage tissue was lost in OA cartilage. **(A,B)** Levels of expression for indicated miRs were measured in knee cartilage tissue isolated from 12 healthy (black) and 15 osteoarthritis (OA, red) donors by qRT-PCR and normalized to U6. Horizontal lines are set at median values. Linear regression is depicted with a solid line, and 95% confidence intervals are depicted with dotted lines in **(B)**; *r*, Pearson correlation coefficient; ***p ≤ 0.001; n.s., not significant.

**FIGURE 7:**
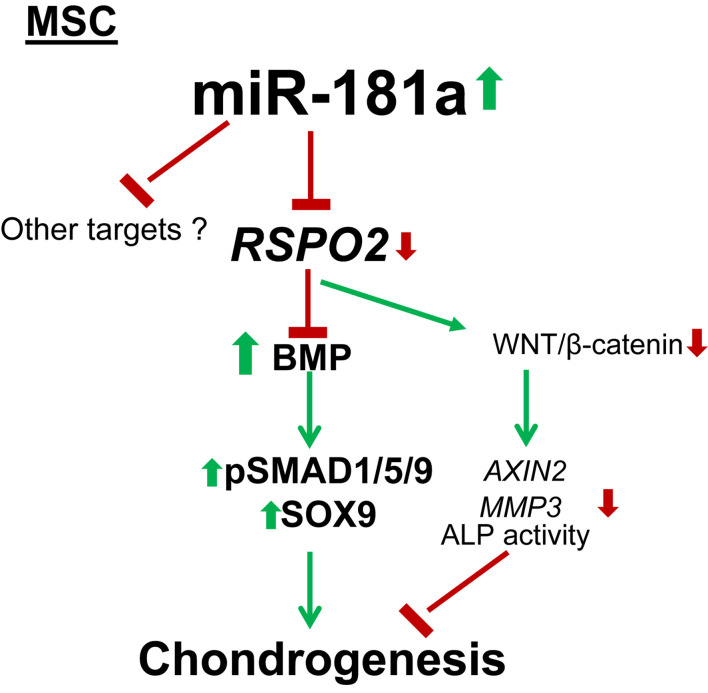
Mechanism for the pro-chondrogenic effects caused by miR-181a. MiR-181a targets *RSPO2* which is a strong agonist of canonical WNT signaling and an inhibitor of BMP signaling. Loss of *RSPO2* results in activation of BMP signaling manifested by increased phospho-SMAD1/5/9 and SOX9 accumulation, as well as in inhibition of canonical WNT target gene expression, *AXIN2*, thus, affecting the balance between WNT and BMP signaling to favor chondral outcome. Thin arrows indicate regulations of the highlighted pathways, and thick arrows display the effects caused by miR-181a; red, downregulation; green, upregulation.

Finally, we were interested to verify whether miR-181a might be aberrantly expressed due to pathological phenotype development of chondrocytes in OA. In contrary to the reports suggesting that an upregulation of miR-181a is a characteristic of cartilage destruction in OA patients (Nakamura et al., 2016, 2019), no substantial differences between healthy and OA cartilage were found for miR-181a in our experimental cohorts. Not only miR-181a expression was not elevated in OA cartilage, but it had the tendency to be at lower levels in comparison with the healthy tissue (Figure 6A). Of note, for stratification of cartilage samples, we have additionally applied expression of miR-675 as a decisive factor to verify the segregation of healthy and OA cohorts (Steck et al., 2012; Hecht et al., 2019; Melnik et al., 2020). Nevertheless, when we tested how miR-181a expression correlates with other hypertrophy-related miRs characterized before (Melnik et al., 2020), we found that there was a tight correlation between miR-181a and miR-218 in healthy tissue. However, this correlation was completely lost in OA samples (Figure 6B). Although the underlying mechanisms regulating biogenesis of these two miRs are unknown, we could hypothesize on possible consequences of the misbalance between miR-181a and miR-218 in OA. For this, we curried out the GO analysis for a combined list of putative mRNA targets for miR-181a and miR-218, to define functional and signaling pathways these two miRs might have in common. We found that targets for both miRs have significant enrichment for genes involved in biological functions GO terms related to signal transduction, protein phosphorylation and differentiation of chondrocytes (Supplementary Table 4). Strikingly, WNT and BMP signaling were among the top 20 consecutive categories on that list. We assessed two additional miRs, miR-31 and miR-210, that earlier have been demonstrated to be differentially expressed between hypertrophic and non-hypertrophic chondrocytes (Gabler et al., 2015). We again observed a similar tight correlation in healthy cartilage between miR-181 and miR-31 that was disrupted in OA. Interestingly, miR-31 has been shown to balance an input from WNT, BMP, and TGF-β signals to coordinate control of intestinal homeostasis, regeneration and tumorigenesis (Tian et al., 2017). In this respect, it highlights an importance of tight regulation of the miR network implicated to WNT and BMP signaling regulation during chondrogenesis that seems to define a healthy cartilage tissue homeostasis and OA prevention. In contrary, another hypertrophy-related miR, miR-210, showed no correlation with miR-181a neither in healthy, nor in OA cartilage tissue. This miR has been earlier implicated to regulation of NF-κB (Ren et al., 2017) and JAK/STAT (Zhang et al., 2020) and WNT (Li et al., 2019), with no evidence for effects on BMP signaling. Collectively, these data indicate that miR-181a regulates the balance between two major signaling pathways, WNT-BMP which define the chondral versus endochondral development in cartilage tissue (Figure 7). Additionally, miR-181a is a likely member of the miR network that define correct WNT-BMP balance that is important for healthy cartilage tissue homeostasis.

## Discussion

This study raises the important question about the role of miRs in establishment and regulation of the hypertrophic phenotype of chondrocytes, – the terminal stage of chondrocyte differentiation that is a prominent characteristic of OA. Hypertrophy is also a very undesirable outcome of MSC chondrogenic differentiation limiting the application of these cells in tissue engineering. The previously published data from our lab identified miR-181a the most prominent miR candidate involved in regulation of different stages of chondrogenesis, however, no precise mechanisms or mRNA targets were established at the time.

Here, we suggest *RSPO2* as a target of miR-181a. *RSPO2* is a member of R-spondin gene family consisting of four genes (*RSPO1/2/3/4*) that encode secreted proteins with high sequence homology and structural similarity. R-spondins are enigmatic secreted proteins that have been found to act as bi-phasic and context-dependent regulators of both canonical and non-canonical WNT signaling (Kazanskaya et al., 2004; Glinka et al., 2011), however, their role in chondrogenesis still remains to be addressed.

A study from the Niehrs group recently reported a new function of R-spondins as BMP receptor antagonists (Lee et al., 2020). Both WNT and BMP signaling are known to synergize in driving chondrocyte hypertrophy (Dexheimer et al., 2016; Diederichs et al., 2019). Hence, this discovery, together with the data presented by our study, implies a new level of regulation where RSPO2 and miR-181a factors might define chondral versus endochondral outcomes as a consequence of their alteration with the WNT-BMP signaling balance. Our study demonstrates that miR-181a can modulate a balance between canonical WNT and SMAD1/5/9 BMP signaling. These effects are in line with the observed significantly reduced levels of *RSPO2* mRNA, as well as with the expected effects on WNT and BMP signaling that would occur due to targeting of *RSPO2*.

We demonstrate that activation of BMP signaling can be uncoupled by miR-181a, resulting in concomitant attenuation of canonical WNT signaling. Thus, these findings reveal that the WNT-BMP network and, therefore, chondral versus endochondral outcome can be manipulated with miR-181a alterations. As no confirmed RSPO2 antibodies suitable for Western blot analysis are available at present, it remains a challenging task to verify RSPO2 as a target of miR-181a at the protein level. Another limitation of the study is that the effects caused by the transient gain of miR-181a achieved with our approach might have lasted no longer than 21 days. However, this time point appears too premature for assessment of the terminal stages of chondrogenesis and the hypertrophic markers, such as ALP activity.

Nevertheless, our findings highlight RSPO2 as a new interesting factor for cartilage biology that might play an important regulatory role in chondrogenic differentiation. Therefore, in future work, more research efforts are necessary in addressing details of its function in cartilage biology.

Collectively, these results demonstrate that miRs represent a novel class of regulatory molecules that can modulate MSC chondrogenesis. However, our data with OA patients clearly indicate that miR-181a cannot act alone in regulating this complex process. Earlier, we identified a whole cluster of stage-specific miRs differentially expressed between articular and hypertrophic chondrocytes during differentiation (Gabler et al., 2015). We also characterized miR-218 as a miR with the potential to suppress multiple hypertrophy-related targets (*RUNX2, MEF2C*, and *COL10A1*) (Melnik et al., 2020). Despite of that, we have also found that reversal of the hypertrophic phenotype cannot be achieved by manipulation of this single miR. Nevertheless, although neither miR-218, nor miR-181a were found to be useful as prognostic markers in OA development in the patient cohorts applied in our study, we found a tight correlation of expression levels for these two miRs in healthy cartilage, which, however, was completely disrupted in OA. This leads to a conclusion that there should be a common mechanism/signaling pathway that regulates expression of these miRs involved in regulation of particular developmental stages during chondrogenesis. Indeed, it has been established before that transcription of miRs involved in the same developmental process is organized at the same transcription factory (Papantonis et al., 2012). Thus, future investigations should be directed to identify the common signaling pathway/transcription factor that regulates expression of the miR cluster network to ensure tight regulation of each miR involved at the particular developmental stage of chondrogenesis. One way to approach this goal would be isolation of primary microRNA (pri-miR)-associated transcription factories at distinct stages of chondrocyte maturation, with defining those key factors that regulate transcription of the particular pri-miR-encoding gene clusters (Melnik et al., 2011, 2016).

## Conclusion

Our findings uncover *RSPO2* as a target of miR-181a and establish a new functional link between miR-181a and modulation of the WNT-BMP signaling crosstalk during MSC chondrogenesis. Therefore, this study will contribute to the development of new approaches that allow manipulation of differentiation outcome of MSC for engineering of neocartilage and for OA pathology intervention in patients.

## Data Availability Statement

The original contributions presented in the study are included in the article/Supplementary Material, further inquiries can be directed to the corresponding author/s.

## Ethics Statement

The studies involving human participants were reviewed and approved by the Medical Faculty of the University of Heidelberg. The patients/participants provided their written informed consent to participate in this study.

## Author Contributions

SM and WR conceived, designed, and supervised the project, and wrote the manuscript. SM and NHo conducted most of the experiments, and processed and analyzed the data. JG produced an initial set of data for the study. NHe performed microRNA analysis from cartilage of donors and analyzed the data. All authors discussed the data, and read, commented, and approved the manuscript.

## Conflict of Interest

The authors declare that the research was conducted in the absence of any commercial or financial relationships that could be construed as a potential conflict of interest.

## Publisher’s Note

All claims expressed in this article are solely those of the authors and do not necessarily represent those of their affiliated organizations, or those of the publisher, the editors and the reviewers. Any product that may be evaluated in this article, or claim that may be made by its manufacturer, is not guaranteed or endorsed by the publisher.

## Footnotes

1
http://www.targetscan.org/vert_72/

2
http://mirwalk.umm.uni-heidelberg.de/

3
https://bioweb.pasteur.fr/packages/pack@miRanda@3.3a

4
https://tools4mirs.org/software/mirna_databases/micrornaorg/
