# Supplementary material for: MiR-181a Targets RSPO2 and Regulates Bone Morphogenetic Protein – WNT Signaling Crosstalk During Chondrogenic Differentiation of Mesenchymal Stromal Cells
Source: Front Cell Dev Biol. 2021 Oct 29;9:747057. doi: 10.3389/fcell.2021.747057 (PMC8586458; doi:10.3389/fcell.2021.747057)
Supplement: Supplementary file 1 [file Data_Sheet_1.pdf]

Supplementary Material

MiR181a targets *RSPO2* and regulates BMP - WNT signaling crosstalk during chondrogenic differentiation of mesenchymal stromal cells

Svitlana Melnik, Nina Hofmann, Jessica Gabler, Nicole Hecht, Wiltrud Richter*

*To whom correspondence should be addressed:
E-mail: wiltrud.richter@med.uni-heidelberg.de

This pdf file includes:

Supplementary Tables S1 – S4
Supplementary Figures S1 – S10

Supplementary Table S1. Summary of clinical data for the healthy cartilage tissue donors and OA patients cohorts used in this study. Severity of OA in each patient was assessed according to the ICRS (International Cartilage Repair Society) scoring. M, male; F, female.

Number	Age	Gender	Disease	ICRS score
1	21	M	Healthy	0
2	35	M	Healthy	0
3	14	F	Healthy	0
4	24	M	Healthy	0
5	30	M	Healthy	0
6	15	M	Healthy	0
7	31	M	Healthy	0
8	16	F	Healthy	0
9	24	M	Healthy	0
10	20	F	Healthy	0
11	38	M	Healthy	0
12	21	M	Healthy	0
1	75	F	Knee OA	4
2	65	M	Knee OA	4
3	73	F	Knee OA	4
4	60	M	Knee OA	4
5	58	M	Knee OA	4
6	78	F	Knee OA	4
7	60	M	Knee OA	4
8	83	F	Knee OA	4
9	62	M	Knee OA	4
10	68	F	Knee OA	4
11	45	M	Knee OA	4
12	52	F	Knee OA	4
13	56	M	Knee OA	4
14	88	F	Knee OA	4
15	55	F	Knee OA	4

Supplementary Table S2. Significantly enriched KEGG pathways associated with the predicted target genes for miR-181a (selected categories for 'Signal transduction' identifiers).

**KEGG pathway Term**	**Genes**	***p*-value**
cGMP-PKG signaling pathway	40	1.8E-6
FoxO signaling pathway	33	3.1E-5
Proteoglycans in cancer	40	4.9E-4
Insulin signaling pathway	30	7.4E-4
Calcium signaling pathway	36	8.9E-4
HIF-1 signaling pathway	23	9.5E-4
TGF-beta signaling pathway	21	9.6E-4
NF-kappa B signaling pathway	21	1.5E-3
cAMP signaling pathway	38	1.5E-3
mTOR signaling pathway	16	1.7E-3
Signaling pathways regulating pluripotency of stem cells	29	2.0E-3
PI3K-Akt signaling pathway	58	2.2E-3
Thyroid hormone signaling pathway	24	4.8E-3
AMPK signaling pathway	25	5.6E-3
WNT signaling pathway	27	6.5E-3
TNF signaling pathway	22	8.6E-3
MAPK signaling pathway	40	3.0E-2
T cell receptor signaling pathway	19	3.3E-2
Hippo signaling pathway	26	3.5E-2
VEGF signaling pathway	13	4.0E-2

Supplementary Table S3 List of cloning primers used in this study

**Name**	**Sequence**
RSPO2_UTR_SpeI-For	cccactagtTGAAACAAAGCAAGGTAAAGCC
RSPO2-UTR-HindIII-Rev	cccaagcttACATCCCAGGAACAACAGGT
RSPO2_mut_For	TATACAACTTTAGGGTTTACATTTAATCCTGAAGTG
RSPO2_mut-Rev	TCATAGCAATATTTTCATAACGATGTATATGATATT

Supplementary Table S4. Gene ontology enrichment for Biological Process category associated with combined lists of conserved target genes predicted for miR-218 and miR-181.

**Biological Process**	**Genes number**	***P* value**
**Signal transduction**	239	4.30E-132
Cell adhesion	120	7.60E-74
Intracellular signal transduction	98	1.60E-56
**WNT signaling pathway**	45	2.30E-25
Extracellular matrix organization	45	1.80E-24
Protein phosphorylation	58	2.80E-18
Peptidyl-serine phosphorylation	30	5.80E-17
Cellular response to hypoxia	26	4.50E-16
Osscification	24	6.30E-16
Embryonic cranial skeleton morphogenesis	16	9.70E-15
Positive regulation of transcription from RNA polymerase II	80	1.40E-13
**BMP signaling pathway**	21	3.50E-13
Homophilic cell adhesion via plasma membrane adhesion molecules	28	2.10E-12
**Canonical WNT signaling pathway**	21	2.10E-12
Positive regulation of osteoblast differentiation	18	5.60E-12
Cell migration	27	9.20E-11
**SMAD protein signal transduction**	17	1.10E-10
Chondrocyte differentiation	14	1.80E-10

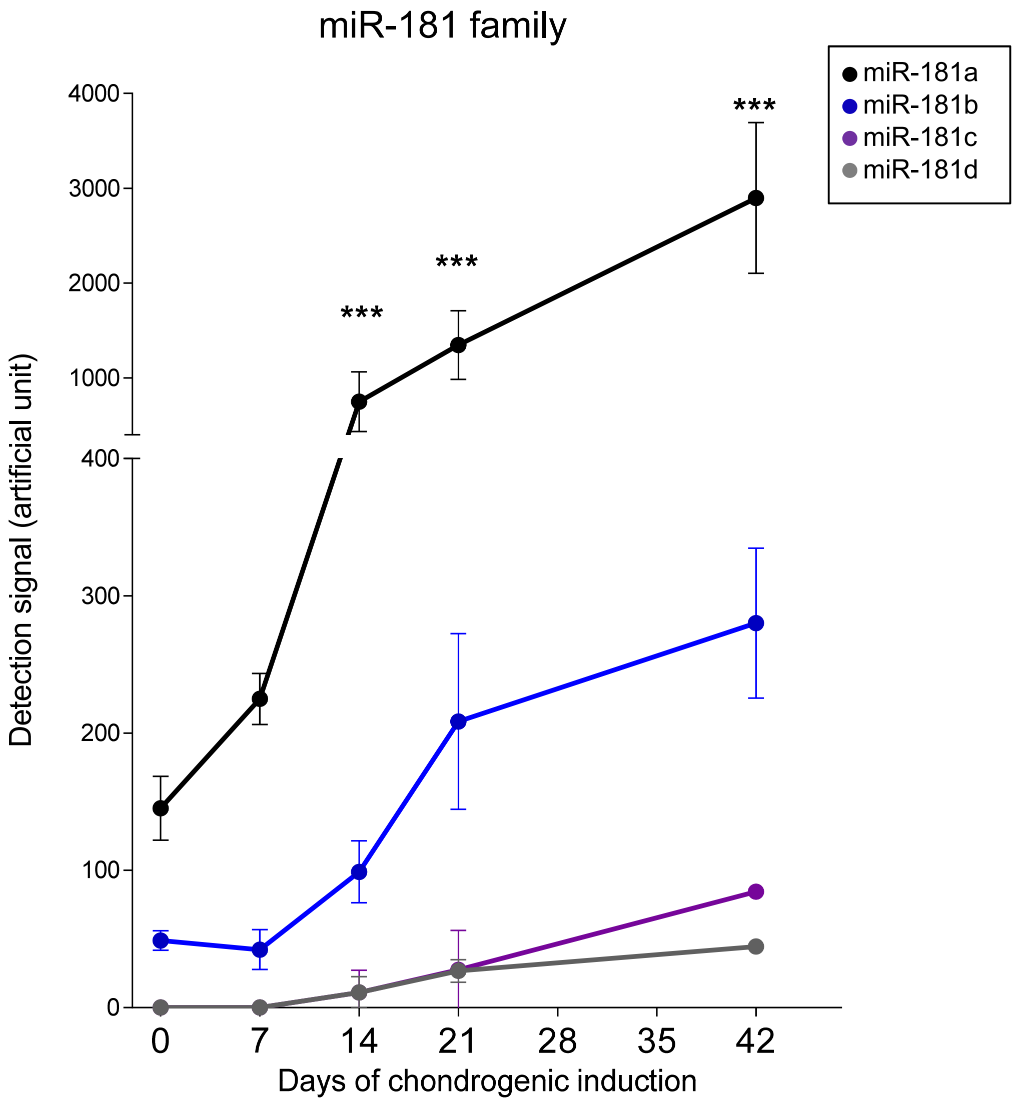

Figure S1. Expression of miR-181 family members (miR-181a (black)/ miR-181b (blue) /miR-181c (purple) /miR-181d (grey)) in MSC (N = 6) monitored at indicated time points during chondrogenic differentiation by microarray analysis; error bars represent mean values ± SD; ***$p \leq 0.001$ (Two-way ANOVA).

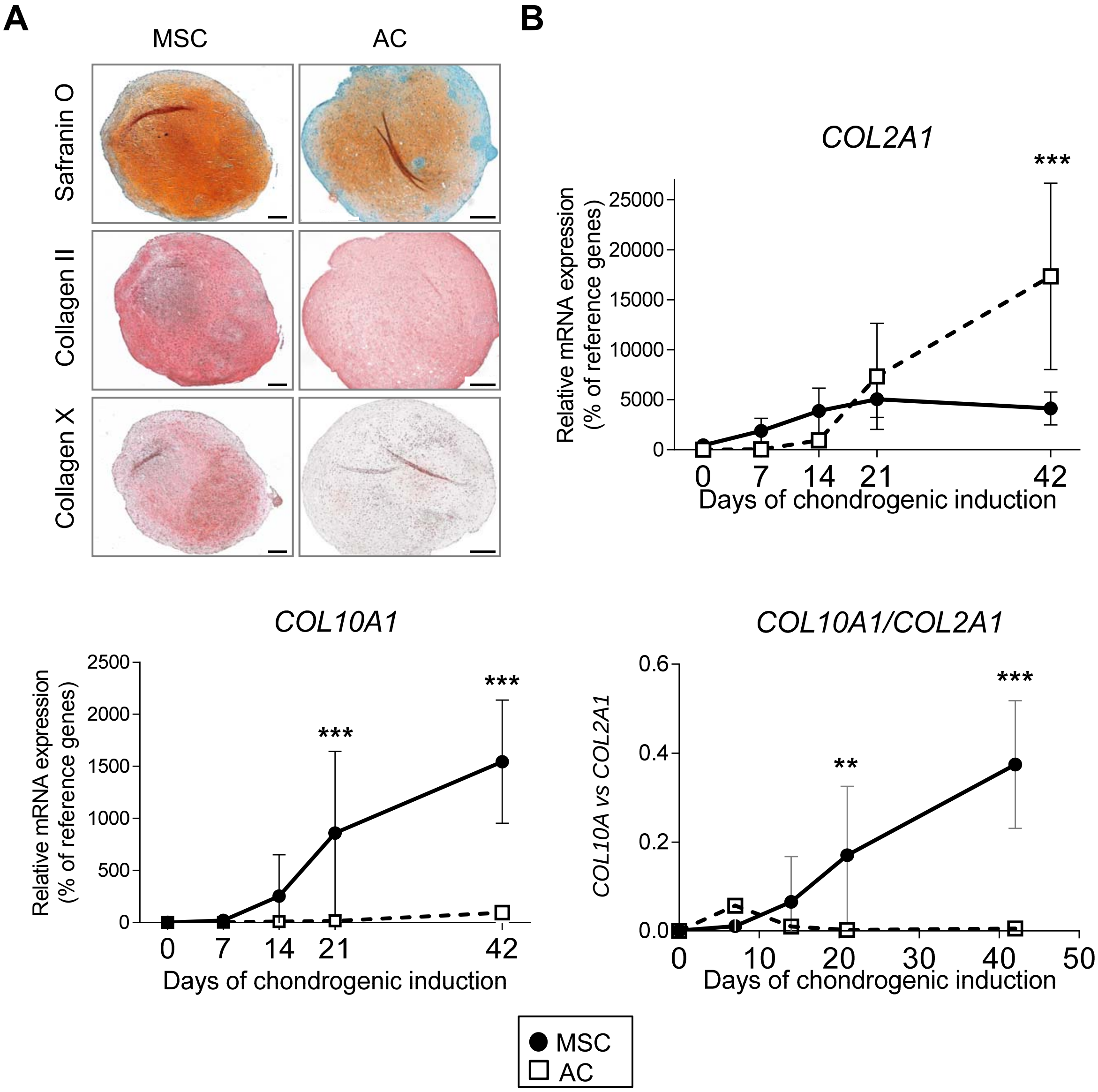

Figure S2. MSC, but not AC, underwent hypertrophy upon chondrogenic induction. MSC (N = 6 donors, black line, black circles) or AC (N = 4 donors, black dotted line, white squares) were subjected to chondrogenic conditions and evaluated at the end of chondrogenesis. **A**, Representative images for Safranin O staining and for collagen type II or type X immunohistochemistry. Scale bar, 100 µm. **B,** mRNA expression for indicated genes was measured by qRT-PCR, and values were normalized to three housekeeping genes (*ACTB*, *HPRT*, *RLP13*). Error bars represent mean values ± SD; ***$p \leq 0.01$, ***$p \leq 0.001$ (Two-way ANOVA).

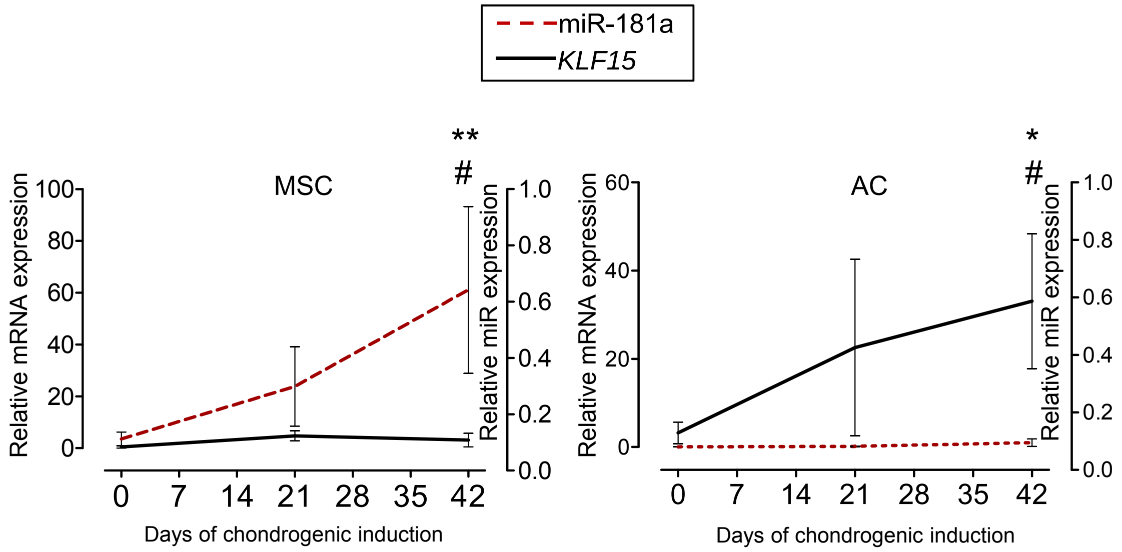

Figure S3. Inverse expression dynamics between *KLF15* and miR-181a in MSC and AC. MSC (N = 6 donors) or AC (N = 4 donors) were subjected to chondrogenic induction, and mRNA expression of *KLF15* (black line) and miR-181a (red dotted line) was measured by qRT-PCR at indicated time points; error bars correspond to mean values ± SD; $**p \leq 0.01$, $*p \leq 0.05$, AC versus MSC at the corresponding time point; $\#p \leq 0.05$, D42 versus D0 for a corresponding mRNA or miR expression (ANOVA test).

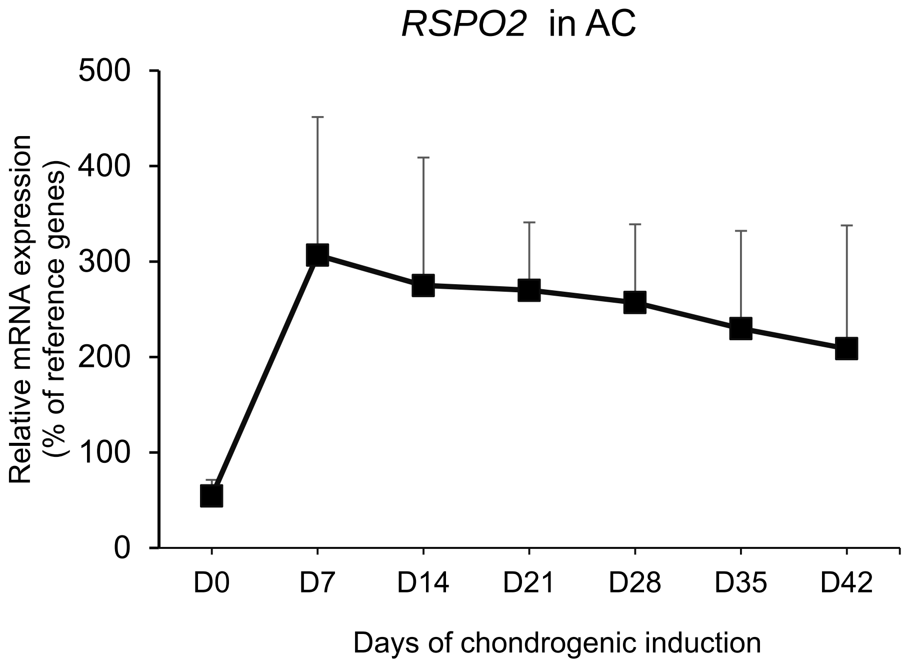

Figure S4. *RSPO2* expression in AC pellet culture during re-differentiation. AC (N = 4 donors) were subjected to chondrogenic re-differentiation, and mRNA expression of *RSPO2* was measured by qRT-PCR at indicated time points; error bars correspond to mean values ± SD.

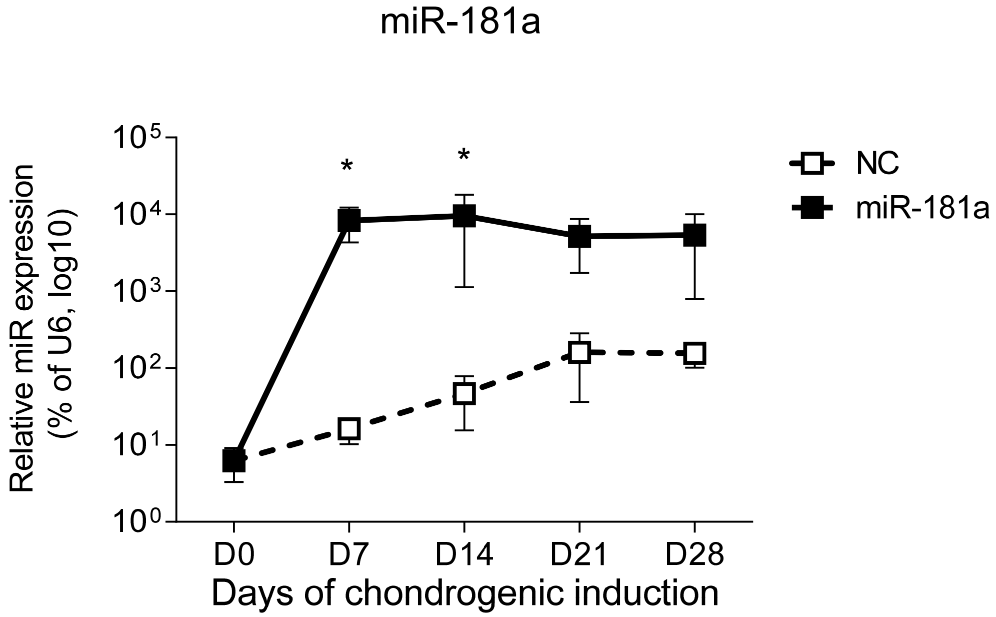

Figure S5. Control experiment to assess the transfection efficiency for miR-181a mimic in MSC which were then subjected to chondrogenesis for 28 days. MSC (N = 3 donors) were transiently transfected with miR-181a (black line, black squares) or with a non-targeting control mimic (NC, black dotted line, white squares), before cells were subjected to chondrogenic differentiation for 28 days. MiR-181a expression was controlled by qRT-PCR at indicated time points; error bars correspond to mean values ± SD; *$p \leq 0.05$ (Two-way ANOVA).

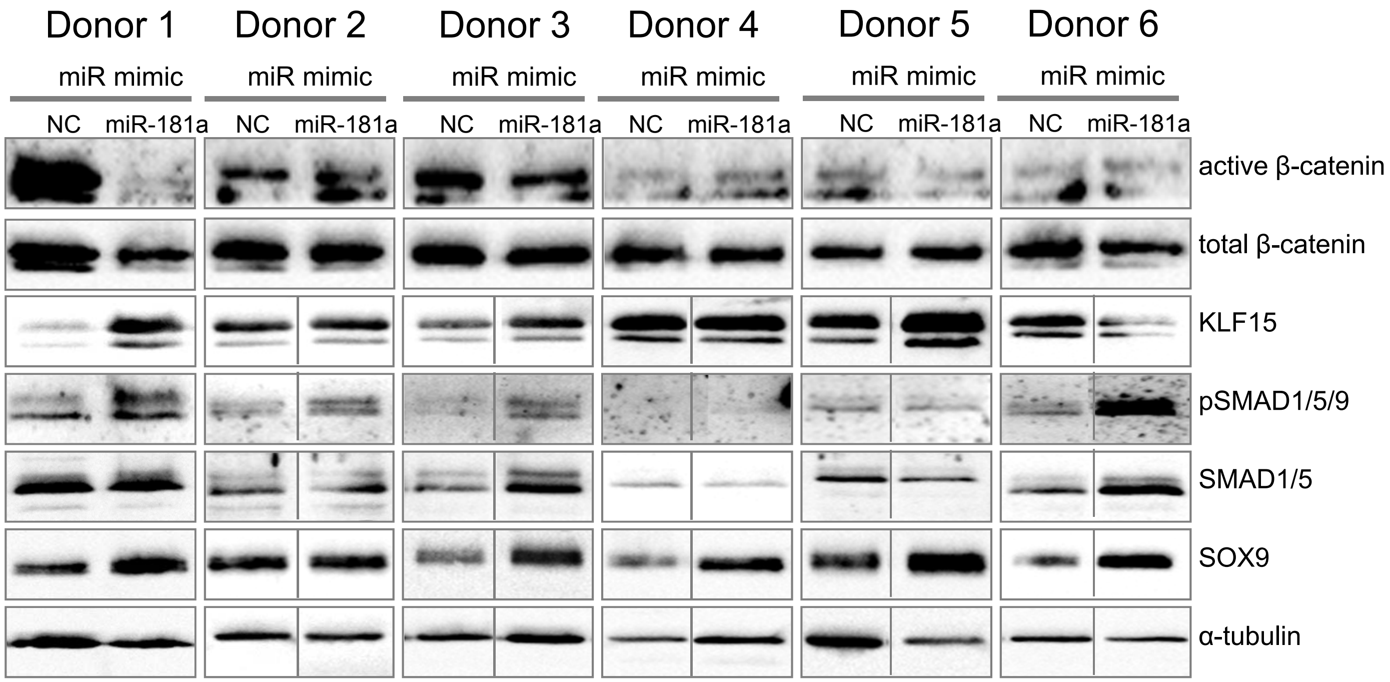

Figure S6. Western blot analysis of 3D pellet cultures at day 28 of chondrogenesis. MSC (N = 6 donors) were transfected with miR-181a or with a control non-targeting miR (NC) mimic at day 0, and accumulation of the indicated proteins was assessed; α-tubulin was used as a loading control.

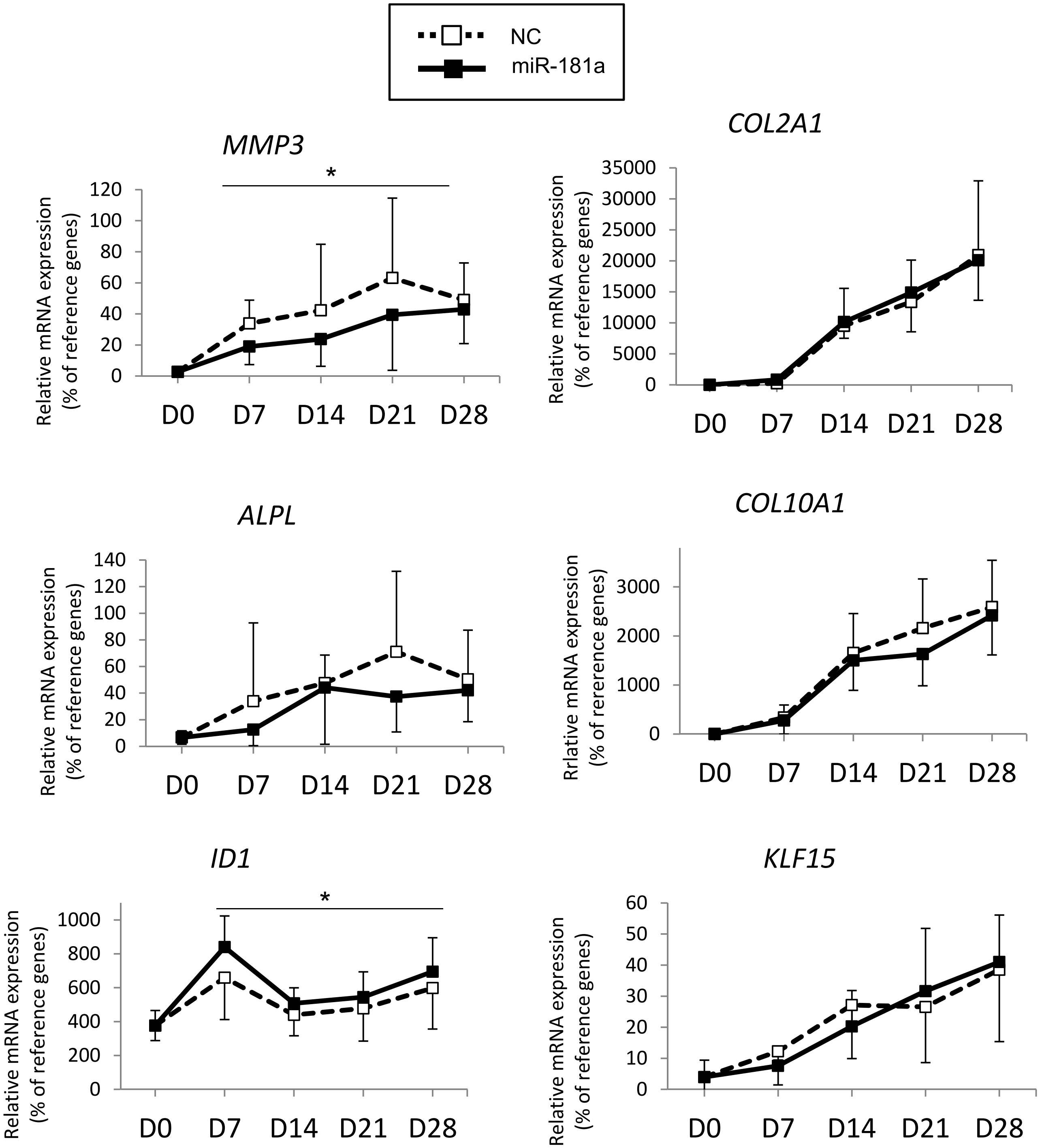

Figure S7. MiR-181a reduced the expression of *MMP3* and *ALPL*, with upregulation of the BMP target gene, *ID1,* in MSC during chondrogenesis. MSC (N = 5 - 6 donors) were transfected with miR-181a (black line, black squares), or with a control non-targeting miR (NC, black dotted line, white squares) mimics at day 0, then subjected to chondrogenic differentiation for 28 days, and mRNA expression for indicated genes was monitored at indicated time points by qRT-PCR. Levels of mRNA expression were normalized to a geomean of *HPRT*, *CPSF6* and *RPL13*; error bars correspond to mean values ± SD. *$p \leq 0.05$, NC vs miR-181a; the expression kinetic curves were compared using paired two-tailed t-test.

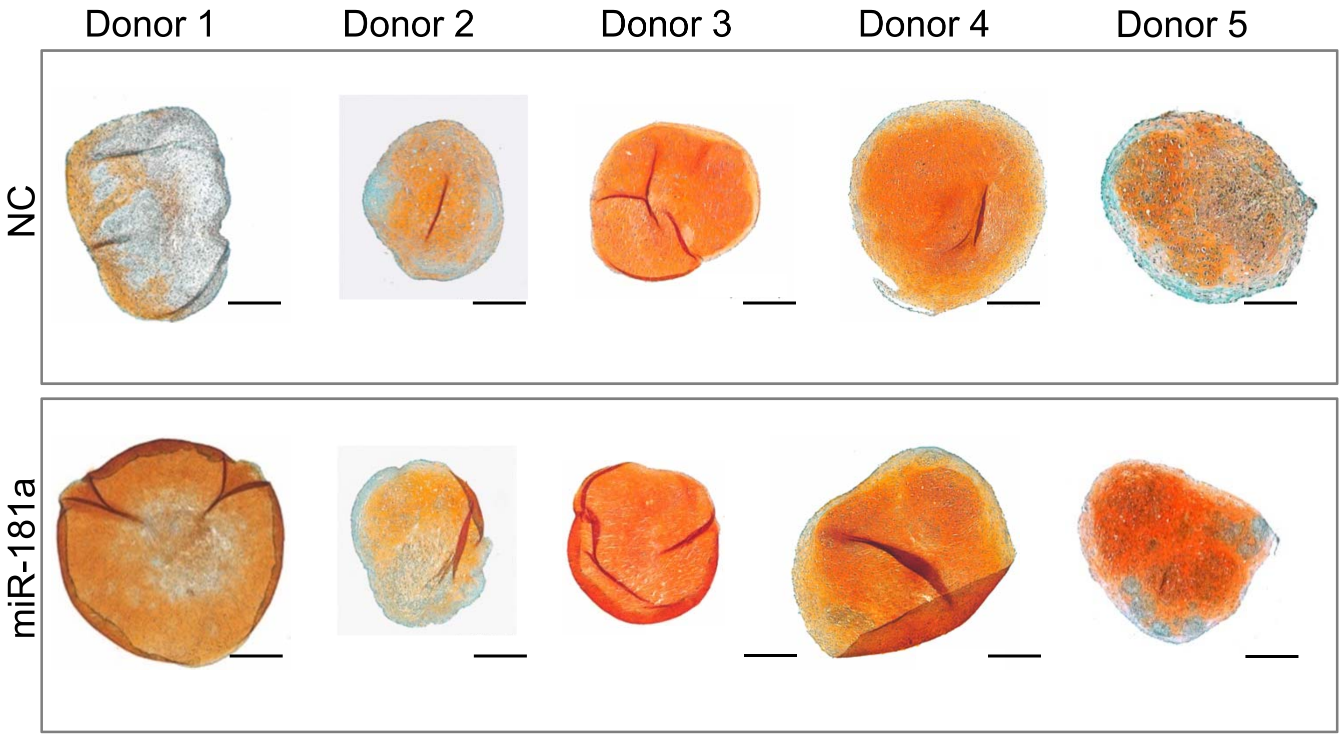

Figure S8. Safranin O staining of 3D pellet cultures at day 28 of chondrogenesis. MSC (N = 5 donors) were transfected with miR-181a or with a control non-targeting miR (NC) mimics at day 0, and subjected to chondrogenic differentiation for 28 days; scale bar, 500 µm.

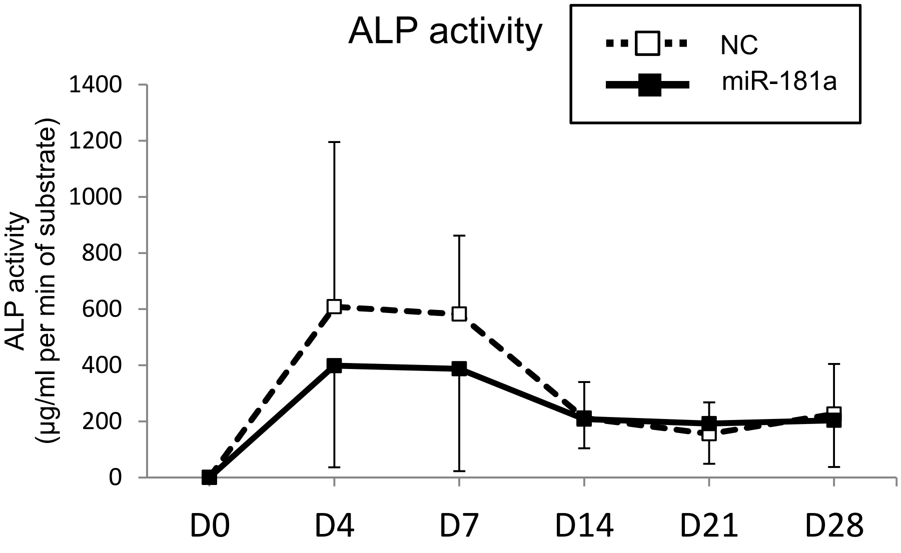

Figure S9. MiR-181a reduced the ALP activity in MSC during chondrogenesis by trend. MSC (N = 7 donors) were transfected with miR-181a (black line, black squares), or with a control non-targeting miR (NC, black dotted line, white squares) mimics at day 0, then subjected to chondrogenic differentiation for 28 days, and ALP activity was monitored at indicated time points; error bars correspond to mean values ± SD.

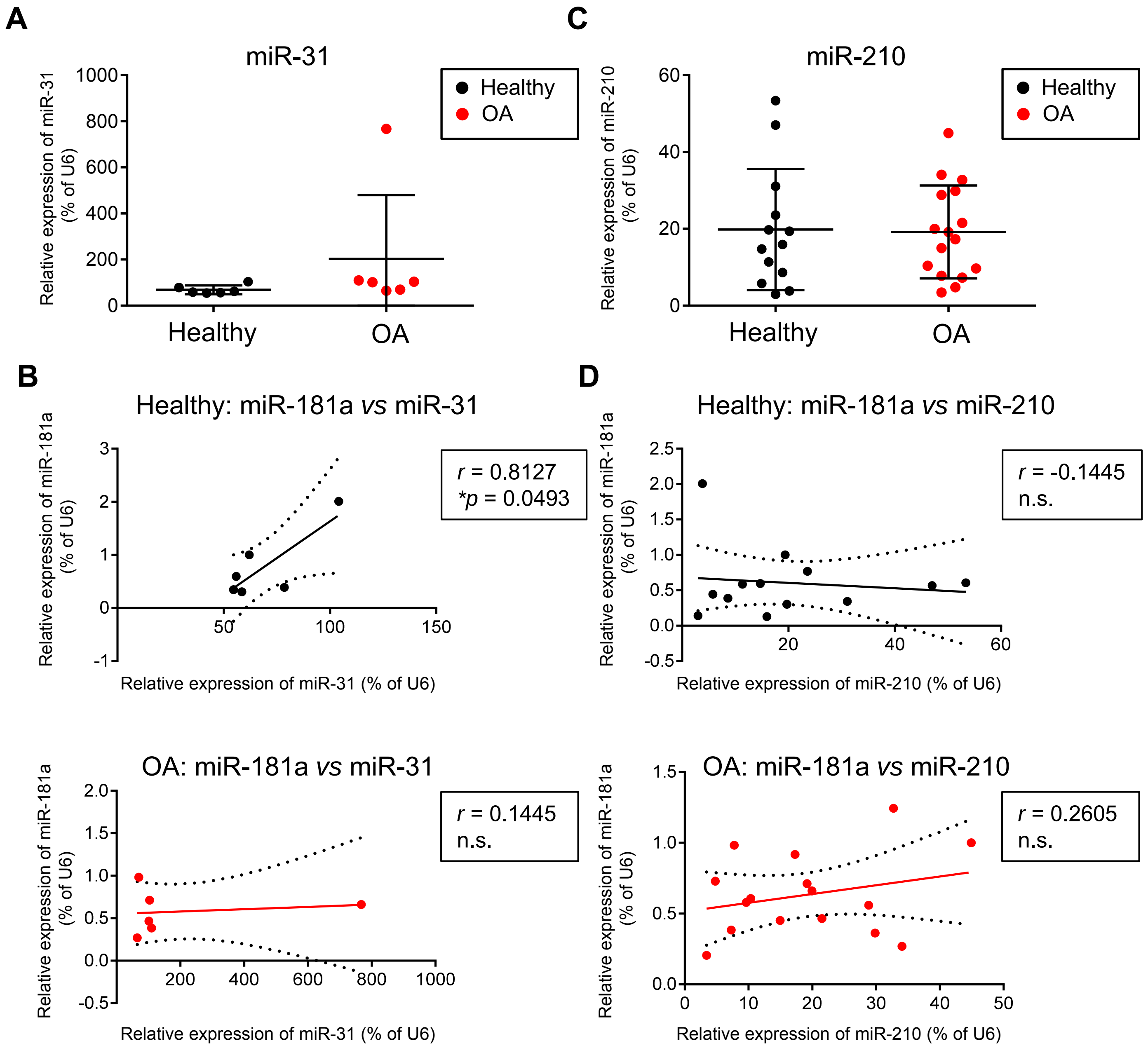

Figure S10. Correlation between miR-181a versus miR-31 in healthy cartilage tissue was lost in OA, and neither healthy nor OA displayed any correlation between miR 181a and miR-210. A-D, Levels of expression for indicated miRs were measured in knee cartilage tissue isolated from 12 or 6 healthy (black) and 15 or 6 osteoarthritis (OA, red) donors by qRT-PCR and normalized to U6. Horizontal lines are set at median values. Linear regression is shown with a solid lines, and 95% confidence intervals are depicted with dotted lines in **B** and **D**; *r* - Pearson correlation coefficient; $*p \leq 0.05$; n.s. - not significant.
